# Veterinary Medical Association of New Jersey

**Published:** 1897-11

**Authors:** S. Lockwood

**Affiliations:** Secretary


Secretary.

### VETERINARY MEDICAL ASSOCIATION OF NEW JERSEY.

THE thirty-ninth regular meeting was held at the State Street House, Trenton, N. J., on October 14, 1897, Dr. Arrowsmith, President, in the chair. Roll called and minutes of previous meeting read and approved. After the regular business of the Association was disposed of it was moved and carried to dispense with the reading of papers at this meeting and devote the time to the preparing of bills to be presented to the next Legislature. Dr. Miller moved the Secretary write a letter of condolence to the family of the late Dr. Rudolph Leis, who was at one time a member of this Association; seconded and carried. Moved and carried that the Secretary present to the Trustees a notice in writing of the proposed changes to the By-laws. Adjourned to meet at Trenton in April, 1898. S. LOCKWOOD,
Secretary.

